# TomoJ: tomography software for three-dimensional reconstruction in transmission electron microscopy

**DOI:** 10.1186/1471-2105-8-288

**Published:** 2007-08-06

**Authors:** Cédric MessaoudiI, Thomas Boudier, Carlos Oscar Sanchez Sorzano, Sergio Marco

**Affiliations:** 1 Institut Curie. Section Recherche. Laboratoire d'Imagerie Intégrative. Centre Universitaire d'Orsay, 91405 Orsay CEDEX, France; 2 INSERM U 759. Centre Universitaire d'Orsay. Bât 112. 91405 Orsay CEDEX, France; 3 Bioengineering Lab, Escuela Politécnica Superior. Univ. San Pablo – CEU. Campus Urb. Montepríncipe s/n. 28668. Boadilla del Monte, Madrid, Spain

## Abstract

**Background:**

Transmission electron tomography is an increasingly common three-dimensional electron microscopy approach that can provide new insights into the structure of subcellular components. Transmission electron tomography fills the gap between high resolution structural methods (X-ray diffraction or nuclear magnetic resonance) and optical microscopy. We developed new software for transmission electron tomography, TomoJ. TomoJ is a plug-in for the now standard image analysis and processing software for optical microscopy, ImageJ.

**Results:**

TomoJ provides a user-friendly interface for alignment, reconstruction, and combination of multiple tomographic volumes and includes the most recent algorithms for volume reconstructions used in three-dimensional electron microscopy (the algebraic reconstruction technique and simultaneous iterative reconstruction technique) as well as the commonly used approach of weighted back-projection.

**Conclusion:**

The software presented in this work is specifically designed for electron tomography. It has been written in Java as a plug-in for ImageJ and is distributed as freeware.

## Background

Elucidation of the three-dimensional (3D) arrangement of subcellular components facilitates understanding of their structure-function relationships and as such is one of the greatest assets of modern biology. With the newest electron microscopes and well-established techniques for sample preparation [1-3], it is increasingly possible to achieve 3D-visualization of subcellular structures in their near-native state at a nanometer resolution [4].

Most 3D-TEM techniques, such as single particle analysis [5] and methods for reconstruction of periodic structures [6] require structurally homogeneous or periodic objects. However, identical molecular conformations are not guaranteed due to the intrinsic and extrinsic variability of biological objects. In addition, although the sizes of crystallized objects are increasing, a large number of macromolecular complexes remain inaccessible to crystallization. Transmission electron tomography (ET) circumvents these limitations, enabling the 3D reconstruction of objects with unique topologies, including most cellular organelles. In addition, ET offers a resolution between that of optical microscopy and that of single particle or periodic structures reconstruction methods [7]. This enables correlative microscopy approaches [8] that combine information from the level of atoms to the level of cells [9].

Computing a volume (tomogram) by ET requires four main steps: acquisition of a series of projections at different tilt angles (tilt series), image alignment, reconstruction, and visualization. The acquisition of tilt series can be performed using different geometries: single-axis [10], dual-axis [11,12], multiple-axis [13], or conical [14,15]. One of the major inconveniences during the acquisition process is specimen shift due to instrumental limitations such as thermal or mechanical instability. These shifts require alignment of the tilt series before reconstruction. Shift correction is accomplished with two types of algorithms, automatic and semiautomatic. Automatic alignment is performed by cross-correlation [16,17], and semi-automatic alignment is performed using fiducial markers [18]. Fiducial markers (frequently gold particles) can also be used to determine the tilt-axis direction around which the tilt series is rotated. Knowledge of the tilt-axis is strictly required for reconstruction. The tomogram is commonly reconstructed using weighted back-projection (WBP) [19], although other algorithms such as the algebraic reconstruction technique (ART) [20] and simultaneous iterative reconstruction technique (SIRT) [21,22] can be used for tomographic reconstruction. Visualization of the computed tomogram is difficult due to the complexity of the data, which necessitates subjective manual segmentation. In order to develop a more objective semiautomatic or automatic segmentation, efforts are underway to denoise data, mostly by application of anisotropic diffusion algorithms [23].

Tomographic reconstructions can be performed using software dedicated for single-particle analysis (such as Spider [24], Imagic [25], Xmipp [26], or EMAN [27]). However, the development of ET [7,28] has been facilitated by freeware specifically designed for ET (first IMOD [18], then TOM [29], EM3D [30], and UCSF tomography [31]. The major limitation of these freeware is that they lack the most powerful reconstruction algorithms, such as ART or SIRT, which are used for reconstruction in single-particle analysis. Here, we present new software for electron tomography, TomoJ, which was developed with the specific intent of offering both an extremely simple interface and a powerful range of algorithms, including automatic and semiautomatic alignment, statistical determination of the tilt axis, and ART and SIRT, as well as the classic WBP, for reconstruction. TomoJ was developed as a Java plug-in for ImageJ [32,33], one of the most frequently used software programs for image analysis. As such, it leverages the extensive capabilities of this freeware for image analysis, such as filters, denoising, and visualization. TomoJ is freely downloadable [49] and has been successfully applied to a variety of biological data sets [34,35].

## Implementation

**Figure 1:**
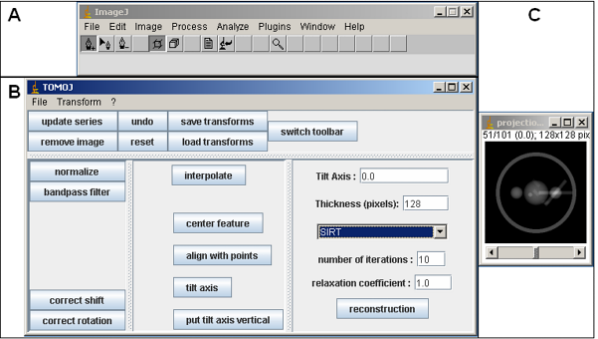
**TomoJ interface**. **A: **ImageJ user interface as presented after launching TomoJ. Note that the toolbar differs from the standard ImageJ toolbar. The standard toolbar can be regained by using the "switch toolbar" button on the TomoJ interface. **B: **TomoJ interface divided into four regions. Top: global menus. Bottom (left to right): the regions devoted to automatic alignment, tools that require point selections, and 3D reconstruction. **C: **Data set window. The header includes the tilt-series name, the number of the visualized image in the total stack, the total number of images in the stack, the tilt angle corresponding to the visualized image, the image byte type, and the total memory occupied by the stack.

TomoJ was implemented as a plug-in for ImageJ software [32]. ImageJ is a well known Java software program developed by the National Institutes of Health; it offers a large number of image analysis capabilities including analysis of electrophoretic bands and multicolor combination of images from confocal microscopy. TomoJ offers the same advantages as ImageJ regarding image analysis, with the addition of easy installation, portability (as a Java-based program, it runs on all operating systems), and simplicity at the user-interface level (Figure 1). In addition, TomoJ can read and write all file formats available in ImageJ as well as the standard electron microscopy image formats MRC [6] and SPIDER [24].

As a plug-in for ImageJ, TomoJ requires this software to be installed from the web [33]. The main limitation of ImageJ, as with any other Java software, is the RAM memory management. ET requires high memory usage, which is limited with 32-bit JAVA processors. Working with large data sets requires 64-bit processors and at least 4 Gigabytes of RAM memory (the procedure for large memory allocation is described in the ImageJ documentation).

Once ImageJ installation is complete, it is simply necessary to copy the distribution file (TomoJ_.jar; downloadable[49] ) to the ImageJ plug-in directory. At this point, for each new execution of ImageJ, a submenu "TomoJ", including two main options, will be available in the plug-in menu. The first option, "TomoJ interface", is used to launch the interface for reconstruction of single-axis tilt series. The second option, "TomoJ average", enables combination of multiple volumes for multiple-axis tomography [13]. TomoJ can also be run from the command line.

### TomoJ workflow

Computation of a single-axis tomogram in TomoJ requires: 1) tilt-angle assignment, 2) tilt-series normalization, 3) tilt-series alignment, 4) tilt-axis determination, and 5) volume reconstruction. All these steps are accessible from a unique interface (Figure 1).

### Assignment of tilt angle

Once a tilt series is loaded into ImageJ, the user is prompted to provide the tilt angle corresponding to the first image and the angular increment used for image recording. Based on these parameters, tilt angles are computed for each image by applying the two most commonly used angular schemes for acquisition: linear or Saxton [36] models. If other acquisition models are used, tilt angles can be provided by a text file. The tilt angles can then be manually modified, if required, for any individual image using the command "assign tilt angle" from the menu of TomoJ.

A single image can be removed from the tilt series using the "remove image" button (Figure 1). This operation does not modify the tilt-angle assignment of other images.

### Normalization of tilt series

Tilt-series images must be placed in a common numerical framework in order to eliminate differences between the individual dynamic ranges. These differences are mainly due to the fact that the electron beam crosses different sample thicknesses as a function of the tilt angle. Normalization also facilitates visualization of the tilt series. Each image (8, 16, or 32 bits) from a tilt series is expanded to a 32-bit real number and normalized to have a mean of 0 and a constant standard deviation (equal to 1), as described in [37] using the equation:

I_n _= (I_m _- Î_m_)/*σ*_m_

where I_n _is the normalized pixel value of an image, I_m _is the original pixel value, and Î_m _and *σ*_m _are the average and standard deviation of the pixels from the whole image, respectively. The normalization procedure is launched by the TomoJ "normalization" button (Figure 1). Output is generated as a 32-bit real image stack.

### Alignment of tilt series

Correct registration (shift and rotation correction) of the images from the tilt series is necessary to produce a quality tomogram. TomoJ enables automatic and semiautomatic alignment. If the data set has a low signal-to-noise ratio, shift and rotation corrections can be computed from filtered data ("band pass filter" button in the TomoJ interface or any other ImageJ filter) and then applied to the original data set ("save transform" and "load transform" buttons).

Automatic shift correction ("correct shift" button, Figure 1) is performed by computing the cross-correlation coefficient (CCC) between every 2 consecutive images and then moving one of the images by the number of pixels required to maximize the CCC. This step is carried out in the frequency domain using the correlation property of the Hartley transform (HT) [38]. The advantage of using the HT is that it requires only half the memory of the most frequently used Fourier transform (FT). In order to preserve the maximum amount of information, the common region is maximized by subtracting the average of all computed shifts between every 2 consecutive images. To improve alignment, a region of interest containing features with high signal-to-noise ratio can be selected. If this region has side lengths of a power of 2, the alignment process is accelerated by using the fast HT. The computed shifts will be applied to the whole stack and can be saved to a text file.

The problem of performing pair-wise comparisons is that, in theory, the final alignment can be quite prone to bias (although in practice this is not always true). Winkler and Taylor [17] have proposed a method for automatic alignment whereby the central image of the tilt series is compared in a pair-wise fashion to its immediate neighbors. Once the neighbors are aligned, they serve as templates for their respective neighbors. This procedure is repeated until all images are aligned. Although this procedure tends to avoid bias, it does not guarantee an unbiased final result. We are currently working on an alignment procedure that avoids alignment bias by considering all images at the same time.

Rotations can be automatically corrected using an equivalent procedure ("correct rotation" button, Figure 1), but if the tilt series was acquired with an automated electron microscope this is usually not required. However, it may be necessary if the tilt series was manually recorded. Rotations between consecutive images are determined by maximizing the CCC in real space or by using the HT power spectrum ("File" menu in Figure 1 and then "options" submenu). Similar to the method for shifts, the average of the computed rotations is subtracted from all rotations.

In the case of important shifts, semiautomatic prealignment ("center feature" button, Figure 1) may be required before automatic alignment. Prealignment requires the user to select point coordinates that correspond to recognizable features present in every image. Selection is performed using the PointPicker's toolbar [39], which replaces the standard ImageJ toolbar on the ImageJ window after launch of the TomoJ interface. The "switch toolbar" button (Figure 1) enables exchange between these toolbars. PointPicker provides a user friendly interactive tool to manually assign coordinates. Once the points are selected, each image is moved so that the barycenter of the feature coordinates is placed at the image center. These precentered images are used as the input for automatic alignment.

If the results of automatic alignment are unsatisfactory, semiautomatic alignment is possible. This method is based on the fact that points belonging to an object rotated along a single axis in 3D space follow arcs around the axis. The 2D projection of the points belonging to these arcs determines parallel lines perpendicular to the tilt axis. As transmission electron microscopy images are projections of 3D objects, the trajectories of any projected point of the 3D object follows lines in the tilt series. The equations of these lines are determined by linear regression of coordinates of recognizable features. Each image is then aligned using the average of the translations needed to project each feature coordinate on its line. The semiautomatic alignment procedure requires knowledge of the feature coordinates on a prealigned tilt series.

### Determination of tilt axis

If the tilt axis is known, then tomogram reconstruction requires only typing the value into the reconstruction panel of the TomoJ window, vertically placing the tilt axis using the corresponding button, and running the chosen reconstruction algorithm. If the image size surpasses the available computer memory, it is possible to obtain a smaller region of interest for reconstruction using selection tools and the "Image->Crop" options from the ImageJ window. This operation requires vertical placement of the tilt axis and switching to the ImageJ toolbar. The cropped region can be located at any position in the stack. After cropping, the user must update the TomoJ image series by clicking the "update series" button (Figure 1). Shift correction in the cropped series is recommended before running the reconstruction algorithms on the cropped region.

If the tilt axis is unknown, it can be determined by 2 procedures. The simplest one, based on the FT of the projected aligned stack, requires discrete and well defined fiducial markers, such as gold particles. In this case, the ImageJ option "Z-project" from the "Image-->Stack" menu enables computation of a projection of the stack using the "min projection" option. From this image it is possible to compute the FT using the "Analysis-->FFT" menu from ImageJ. Finally, the tilt angle can be measured using the angle tool of the ImageJ window. The tilt angle is defined by the vertical line crossing the center of the FT power spectrum image shown on the screen and the tilt line that appears on it.

The second procedure uses the coordinates of a set of selected points from recognizable features or fiducial markers. The features coordinates are used to compute by linear regression the equation of the line perpendicular to the tilt axis. This line represents the average direction of the features displacements. Before their use for tilt-axis determination, features coordinates are validated using the Pearson test to fit linear trajectories. The Pearson test is performed on the linear regression coefficients for each feature. The features not belonging to their line equation with a confidence of 0.95 are excluded from further calculations. The tilt axis is computed as the line perpendicular to the average feature displacement. The software provides the angle (in degrees) required to position the tilt axis vertically. In addition, a confidence interval for the tilt angle is computed from the confidence interval for the slope of the average feature displacement equation. This method requires the presence of at least three recognizable features in most of the tilt-series images. At this stage, it is possible to mark a feature in some of the images (for example, every 10 images) using the PointPicker toolbar and to interpolate the rest of the points using the "interpolate" button. The position of the interpolated points can be corrected with the "move crosses" tool from the PointPicker toolbar. Once one object is selected along the whole stack, the operation can be repeated with the next object. At any step, the tilt axis can be computed by clicking on the corresponding button of the TomoJ window. The value of the tilt axis will be shown in a results window with its corresponding statistical error. Depending on the computed error, the user can complete the tilt-axis determination procedure or choose between correcting the point positions or adding new points. The points can also be used to align the images using the "center with points" option from the TomoJ menu. However, in most cases this operation is unnecessary because the images have previously been centered. Point coordinates can be saved as a text file and retrieved at any moment using the "save points" option from the PointPicker toolbar.

### Reconstruction

The final step in computing a tomogram is reconstruction. For this purpose, the image stack must be aligned and the tilt axis determined. Then, it is necessary to indicate the volume thickness that corresponds to the expected number of voxels that the reconstruction will occupy in the Z-direction. The sample thickness in pixels can be approximated by multiplying the sample thickness in nanometers by the sampling size in nm/pixel.

Three reconstruction algorithms are implemented in TomoJ: WBP [40], ART [20], and SIRT [21,22]. WBP is the most commonly used algorithm for reconstruction in ET. This method compensates for overemphasis of low frequencies in Fourier space by using a weighting scheme prior to reconstruction. Subsequently, the aligned and weighted projections are back-projected into a 3D volume using bilinear interpolation. ART and SIRT are performed in an iterative manner so that the projections of the reconstructed volume computed through an image formation model resemble the experimental projections obtained by the microscope. A linear projection model with additive Gaussian noise is assumed [21]. The linear projection model is a first-order approximation of the nonlinear image formation process occurring in the microscope [41,42]. Furthermore, despite the fact that the noise is not white [43], the SIRT formula for white noise is known to produce good estimates of the underlying structures [44].

No special inputs are required to compute a volume using WBP in TomoJ. For the iterative algorithms ART and SIRT, it is necessary to provide the number of iterations and the relaxation coefficient. In our experience, usually at least four iterations are required to obtain a good tomogram. The choice of the number of iterations depends on the mean square error computed from the differences between the projections of the reconstructed volume and the experimental data. An error curve showing the error as a function of the iteration number is displayed after completing the reconstruction process. The second parameter, the relaxation coefficient, is a weighting factor used to improve the quality of reconstruction, usually at the expense of convergence [45]. TomoJ proposes for ART a relaxation coefficient of (Number of iterations)^-1^, and of 1 for SIRT, which experimentally are good values for most cases. However, these values can be manually modified. In general, lower relaxation coefficients should be used with data sets having a low signal-to-noise ratio, and higher values in the opposite case. The error curve also assists in the choice of the relaxation coefficient: rapid convergence indicates that the relaxation coefficient is too high and slow convergence indicates that it is too low.

Use of all the TomoJ tools is not strictly required to obtain a tomogram. For instance, the tilt-axis angle is provided by most microscope acquisition software. Thus, it can be directly provided by the user. In addition, rotations are normally negligible in an automatic acquisition procedure. Therefore, in most cases only the "normalize", "correct shift", "put tilt axis vertical", and "reconstruction" steps are needed.

### Volume combination in multiple-axis tomography

The inability to record projections at all tilt angles during acquisition results in incomplete information. This inability is due to the increase in the effective sample thickness (T) as a function of the initial thickness (t) and the tilt angle (*α*): T=tcos⁡(α)
 MathType@MTEF@5@5@+=feaafiart1ev1aaatCvAUfKttLearuWrP9MDH5MBPbIqV92AaeXatLxBI9gBaebbnrfifHhDYfgasaacH8akY=wiFfYdH8Gipec8Eeeu0xXdbba9frFj0=OqFfea0dXdd9vqai=hGuQ8kuc9pgc9s8qqaq=dirpe0xb9q8qiLsFr0=vr0=vr0dc8meaabaqaciaacaGaaeqabaqabeGadaaakeaacqWGubavcqGH9aqpdaWcaaqaaiabdsha0bqaaiGbcogaJjabc+gaVjabcohaZjabcIcaOGGaciab=f7aHjabcMcaPaaaaaa@37E0@, implying an infinite thickness to traverse at *α *= 90°. This lack of information is known as the missing wedge [10]. The missing wedge induces reconstruction artifacts (such as the elongation of patterns) in the reconstructed volume perpendicular to the horizontal plane. To compensate for this lack of information, it is possible to rotate the sample in the horizontal plane, recovering a portion of the missing projections. In this way, if two rotations (usually 0° and 90°) are used to record two tomographic series (each series with its own maximum tilt angle), and the two reconstructed volumes are combined in a merged tomogram, then the missing wedge is transformed into a missing pyramid. This approach is referred to as dual-axis tomography [11,12]. An extension of this approach is to record tomographic series at the maximum possible number of rotations in the horizontal plane, which results in a missing cone. This approach is referred to as multiple-axis tomography [13]. Both dual- and multiple-axis reconstructions require alignment of volumes before computing a final average. TomoJ provides a second user friendly plug-in for combining the different single-axis reconstructions by simply averaging volumes after registration.

### Running TomoJ in batch mode

Some of the tasks for computing or merging tomograms are greatly time consuming, depending on the processor and memory used. As a plug-in for ImageJ, TomoJ runs in the user window, which can block the computer from performing other interactive tasks. To overcome this problem, TomoJ can be run in batch mode. This can be accomplished using various commands from the terminal; the full set is provided in the user manual (available from the TomoJ web page).

## Results and Discussion

**Figure 2:**
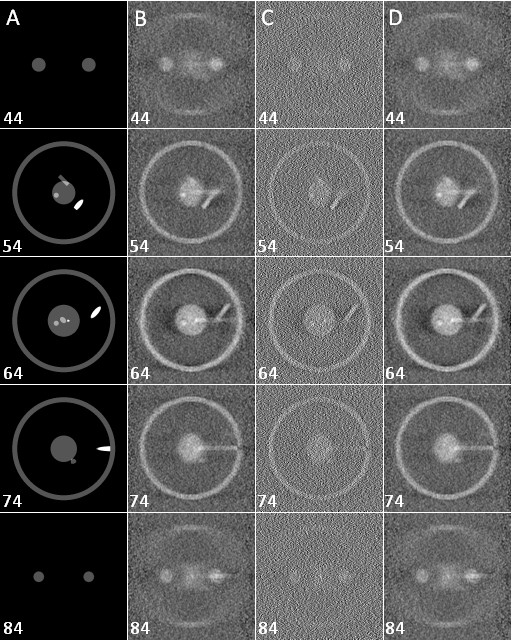
**Comparison between phantom data and reconstruction**. Slices 44, 54, 64, 74, and 84 of every volume are shown. Reconstructions were performed on noisy projections of a phantom (range: -50° to +50°, every 1°, vertical tilt axis). **A: **Original phantom. **B: **Reconstruction using the ART with 10 iterations and a relaxation coefficient of 0.01. **C: **Reconstruction using WBP. **D: **Reconstruction using the SIRT with 10 iterations and a relaxation coefficient of 1.

TomoJ enables 3D reconstruction from tilt-series recorded around a tilt axis, as in ET. The main advantage of this software is the availability of different algorithms for reconstruction. Thus, unlike other public domain software for electron tomography reconstruction (IMOD [18], TOM [29], EM3D [30], and USCF tomography [31]) TomoJ can perform 3D reconstruction using the ART [20] and SIRT [21]. The use of the ART and SIRT provides better results than standard WBP [40] implemented in other software. This is due to the fact that the two main limitations of electron tomography are the missing wedge and the low signal-to-noise ratio of the images (this is mainly the case with cryo-tomography). The robustness of WBP, ART and SIRT has been evaluated by comparison of the 3D reconstructions computed by these three methods to an original phantom. Such comparison can be performed using the coefficient of determination (COD) [22,46]. The COD is nearer to 1 when objects are more similar and nearer to 0 when objects are more different. Running TomoJ on the projections of the phantom shown in Figure 2a, after addition of Gaussian noise to the projections (standard deviation of 25 in order to better simulate the images obtained at the electron microscope), we obtained a COD of 1.5%, 25.4%, and 25.6% for WBP, ART, and SIRT, respectively. The COD obtained using WBP and IMOD (2.2%) was equivalent to that for TomoJ WBP (1.5%). Figure 2 shows the greater accuracy with ART and SIRT versus WBP.

In addition to offering different reconstruction algorithms, TomoJ leverages the capabilities of ImageJ for preprocessing and denoising images. Different image filters and denoising procedures, such as anisotropic diffusion, can be used by downloading the corresponding plug-ins from the ImageJ web page. These features are unavailable in other tomographic freeware.

ImageJ also provides tools for visualization (such as the plug-in VolumeViewer). Therefore, the development of specific visualization tools inside TomoJ is not required. In addition, ImageJ offers segmentation tools such as manual tools and snake or watershed algorithms to assist interpretation of complex volumes, which requires segmentation before visualization. Using the TomoJ export options (MRC and SPIDER, appearing in the "save as" menu of ImageJ after TomoJ installation), the segmented volumes can also be exported to optimized freeware, such as Chimera [47], for visualization. The main advantage of Chimera is that it provides the required functionality for visualization of volumes and is the standard for visualization of macromolecular 3D reconstructions from electron microscopy data.

As Java-based public domain software implemented as a plug-in for ImageJ, TomoJ can be used for research as well as instruction. Increasingly, cell biology laboratories are becoming interested in electron tomography. The user-friendly interface of ImageJ, the easy installation of TomoJ, and the multiplatform capability (TomoJ has been successfully tested on different architectures: Bi-Opteron 250, with 8 or 16 Gb RAM running Linux fedora core; Windows XP 64; and PCs with Intel Pentium IV and Centrino Core 2 duo with 1–2 Gb RAM) facilitate use of TomoJ in biology laboratories. In addition, TomoJ includes algorithms for multiple-axis tomography [13], which allows tomographic reconstruction without electron microscopes equipped with high-tilt devices. These microscopes, which are not devoted to tomography, are available in most electron microscopy services in research centers and universities. In addition, TomoJ will be expanded to compute 3D chemical mapping. This will enable calculation of the spatial distribution of chemical elements from tilt series acquired with the electron microscope at different energy-loss values [48]. Calculation of these chemical maps requires background subtraction using specific software (EFTETJ) [49], which has already been developed as a plug-in for ImageJ in a preliminary version. The EFTETJ output can be used as an input for TomoJ to compute the 3D chemical map.

## Conclusion

In this paper, we present new software for electron tomography. This software was specifically designed to be user-friendly, and it encompasses all algorithmic steps needed for tomographic reconstruction. It also implements the most recent algorithms for tomographic reconstructions. The package has been written as a plug-in for ImageJ and benefits from all the possibilities offered by this freeware. As a Java program, it runs on all JAVA platforms. TomoJ can be freely downloaded [49].

## Availability and requirements

**Project name: **TomoJ

**Project home page: **

**Operating system: **platform independent

**Programming language: **Java

**Other requirements: **Java 1.5.0 or higher

**License: **Cecill

**Any restrictions to use by non-academics: **licence needed

## Authors' contributions

CM carried out the design and implementation of the software. TB participated in the design and implementation of the reconstruction algorithms. COSS participated in the design of the reconstruction algorithms. SM conceived and coordinated the study, and participated in its design. All authors read and approved the final manuscript.
